# Examining the challenge-hindrance-threat distinction of job demands alongside job resources

**DOI:** 10.3389/fpsyg.2023.1011815

**Published:** 2023-02-17

**Authors:** Martha Fernandez de Henestrosa, Philipp E. Sischka, Georges Steffgen

**Affiliations:** Department of Behavioral and Cognitive Sciences, Faculty of Humanities, Education and Social Sciences, Esch-sur-Alzette, Luxembourg

**Keywords:** job demands-resources model, challenge-hindrance-threat model, burnout, work-engagement, well-being

## Abstract

**Purpose:**

Previous research conducted on the Job Demands-Resources model has mostly ignored the newly introduced Challenge-Hindrance-Threat distinction of workplace stressors. Thus, to better understand the nature of job demands, the present study aimed to explore this distinction of job demands within the framework of the Job Demands-Resources model. Moreover, it examined competing theoretical frameworks by investigating the associations between job characteristics and psychological health variables (i.e., burnout, vigor).

**Design/methodology/approach:**

Data were collected *via* computer assisted telephone interview among a representative sample of employees working in Luxembourg (*n* = 1,506).

**Findings:**

Structural Equation Modeling supported the distinctiveness of the proposed demand categories in terms of their effects. The health impairing nature of threats, hindrances, and challenges, as well as the motivational potential of resources was supported. Yet, scarce support was found for the moderating effects of demands and resources on employees’ well-being.

**Research implications:**

Based on these findings, we argue for an extended framework of job characteristics, which will more accurately describe their nature and effects on employees.

**Practical implications:**

In order to promote employee’s well-being, occupational health advisors need to be aware of the distinct demand-wellbeing relations when implementing job redesign measures.

**Originality/value:**

Combining multiple theoretical frameworks is considered a leading principle in occupational health research. The present study implements an extended classification framework of workplace stressors into one of today’s most influential theoretical framework of job characteristics.

## 1. Introduction

Over the last decades, scholars conducting research on the topic of occupational health/workplace stress have often relied on influential theoretical frameworks to establish a link between job characteristics and employees’ health (Eurofound and EU-OSHA, 2014). The Job Demands-Resources model (i.e., JD-R; Bakker and Demerouti, 2017) is one pertinent example for such an approach. Since its introduction in the 2000s, it has been subject of continuous development (Bakker and Demerouti, 2017). While early versions of the model categorized health impairing job characteristics as job demands, and stimulating characteristics as job resources, studies conducted within later stages of the model pointed toward the idea that the nature of job demands may not always be as homogenous as initially contemplated (e.g., Van den Broeck et al., 2010). That is, some job demands may also have positive effects for employees and institutions (e.g., Crawford et al., 2010). In reply, researchers have predominantly differentiated demands into hindrances (i.e., work-related demands constraining employees) or challenges (i.e., work-related demands associated with potential gains for employees; Searle and Tuckey, 2017; O’Brien and Beehr, 2019).

Yet, in recent years, a new differentiation of workplace stressors emerged. Although research on the Challenge-Hindrance-Threat model (C-H-T) of workplace stressors is still in its infancy (Tuckey et al., 2015; Searle and Tuckey, 2017; Tuckey et al., 2017), studies conducted on this framework have particularly failed to consider job resources when statistically examining the link between these stressor types and employees’ well-being. Yet, job resources are defining features to understand stress (O’Brien and Beehr, 2019). To that end, the present study aimed to examine the novel differentiation of workplace stressors while simultaneously taking into account the concept of job resources. Specifically, the present study investigated (a) whether a threefold differentiation of job demands may better represent the nature of job demands than a twofold differentiation, or a one-fold understanding of job demands, and (b) whether different types of demands may have differing relations with burnout and vigor, while simultaneously controlling for the effects of job resources. In addition, we explored (c) whether interactive effects may emerge between job resources and different types of job demands.

## 2. Literature review and research hypotheses

### 2.1. Literature review

#### 2.1.1. The early job demands-resources model

According to the JD-R model, every occupation is characterized by job characteristics that are related to employees’ well-being (Bakker and Demerouti, 2017). In doing so, early developments of the model broadly distinguished between two types of job characteristics, namely job demands and job resources (Bakker and Demerouti, 2017). Job demands are defined as work aspects that demand constant effort and are linked to physiological and psychological costs (Bakker and Demerouti, 2017). In contrast, job resources are defined as work aspects that (a) help employees to accomplish their work goals, (b) promote personal growth and development, and (c) buffer the adverse effects of job demands (Bakker and Demerouti, 2017). While job characteristics such as work-overload, high work-pressure and emotional demanding interactions constitute examples of job demands (Demerouti and Bakker, 2011; Bakker and Demerouti, 2017), autonomy and social support are prominent job resources (Schaufeli and Taris, 2014). Specifically, job demands are expected to lead to employees’ exhaustion and work-related burnout (i.e., health impairment process), while job resources are considered to promote engagement and performance (i.e., motivational enhancing process; Bakker and Demerouti, 2007). Several cross-sectional (e.g., Bakker et al., 2003) and longitudinal studies (e.g., Hakanen et al., 2008) have provided evidence for the dual mechanisms of these job characteristics.

#### 2.1.2. The amended job demands-resources model

Note that while early research conducted on the JD-R model differentiated between work-related risk (i.e., job demands) and resilience factors (i.e., job resources), no qualitative differentiations were made within the category of job demands (Searle and Tuckey, 2017). Thus, all job demands were viewed as solely harmful for employees (i.e., having equal effects on well-being outcomes; Searle and Tuckey, 2017). Yet, later developments of the JD-R model adopted a more differentiated approach by distinguishing between job hindrances and job challenges (e.g., Crawford et al., 2010; Van den Broeck et al., 2010). In this regard, job hindrances are defined as “work circumstances that involve excessive or undesirable constraints that interfere with or hinder an individual’s ability to achieve valued goals” (Cavanaugh et al., 2000, p. 67). They are expected to elicit negative emotions among employees, to act energy depleting, and therefore, to positively associate with unfavorable outcomes, such as burnout and turnover (Van den Broeck et al., 2010; Tuckey et al., 2015). Prominent examples of this demand category are concerns with job security (Cavanaugh et al., 2000) and role ambiguity (Tuckey et al., 2015). Being present in most work environments and experienced across different occupations (Wiegand et al., 2012; Brady and Cunningham, 2019), both job hindrances have been found to have negative consequences for employee’s attitudes and their health (Rizzo et al., 1970; Sverke et al., 2002; Schmidt et al., 2014). In contrast, job challenges are referred to as “work-related demands or circumstances that, although potentially stressful, have associated gains for the individuals” (O’Brien and Beehr, 2019, p. 963). In this sense, they are expected to (a) enable growth and development (i.e., intrinsic gains) and (b) help employees to achieve their goals (i.e., extrinsic gains; Van den Broeck et al., 2010; O’Brien and Beehr, 2019). Prominent examples of job challenges are time pressure and mental demands (Cavanaugh et al., 2000; Tuckey et al., 2015). They have been found to positively associate with different components of work-engagement (Bakker et al., 2005a; Mauno et al., 2007). Yet, job challenges are also considered to act in an energy depleting manner, and are therefore expected to associate positively with ill-being outcomes (e.g., burnout; Crawford et al., 2010; Bakker and Demerouti, 2017).

#### 2.1.3. The challenge-hindrance-threat model

Although the Challenge-Hindrance (C-H) framework has been useful to classify a plethora of workplace stressors, and therefore, to explain their effects (Espedido and Searle, 2018; Brady and Cunningham, 2019), scholars have recently questioned this twofold categorization (e.g., Mazzola and Disselhorst, 2019; LePine, 2022), as it may not accurately account for all features inherent within the stress process itself (Tuckey et al., 2015; Searle and Tuckey, 2017). In this regard, Tuckey et al. (2015) expanded the understanding of the nature of workplace stressors by introducing the concept of job threats. Job threats are defined as “work-related demands or circumstances that tend to be directly associated with personal harm or loss” (Tuckey et al., 2015, p. 6). The authors referred to workplace aggression (i.e., bullying, harassment), customer related social stressors and emotional labor as pertinent examples for job threats (Tuckey et al., 2015). Specifically, workplace mobbing has been identified as a major occupational stressor, resulting in reduced employees’ health, among others (Lee and Brotheridge, 2006). Moreover, it has been linked to severe personal effects (e.g., burnout; Nielsen and Einarsen, 2012). In a similar vein, emotional demands have been linked to different ill-being outcomes (i.e., emotional exhaustion, Zapf, 2002) and are presumed to alienate employees from their emotions, resulting in feelings of inauthenticity (Tuckey et al., 2015). That said, job threats are presumed to go beyond job hindrances. While job hindrances are expected to prevent employees from goal attainment, resulting in feelings of frustration (Tuckey et al., 2015), job threats are presumed to elicit feelings of fear and anxiety, which blend into the actual experience of harm or loss (Lazarus and Folkman, 1984). Note that the difference between hindrances and threats is argued to center on their qualitative different outcomes (i.e., effects) and on the way they are handled (Tuckey et al., 2015). Whereas individuals may successfully overcome hindrances, resulting in positive outcomes, the prevention of the threat stressor itself and its negative effects may constitute the best possible result (Tuckey et al., 2015). Importantly, first empirical findings have provided support for Tuckey et al. (2015) extended understanding of job demands. For example, a qualitative study among retail workers provided an in-depth insight into the threefold conceptualization of workplace stressors by elucidating perceived resource shortfalls and resource uses linked to every type of job demand (Tuckey et al., 2017). An experimental study among undergraduate students examined the differential relations of threats and hindrances on performance outcomes (Espedido and Searle, 2018). Moreover, a recent cross-sectional study among full-time workers and students working part-time successfully included the concept of threat stressors when examining common psychosocial workplace stressors (Brady and Cunningham, 2019). Yet, most research conducted within the field of occupational health continues to apply the C-H perspective to the examination of work-related stressors, equalizing hindrances with threats (Tuckey et al., 2015; Espedido and Searle, 2018). Note, however, that there has been a recent call to extend research on the C-H framework to include threatening demands (LePine, 2022). In reply, the present study seeks to address this concern by integrating the concept of job threats into the JD-R model, resulting in a more extended classification of job characteristics into threats, hindrances, challenges, and resources.

#### 2.1.4. The importance of job resources

First studies conducted on the C-H-T framework have failed to consider job resources when statistically examining the link between these three stressor categories and diverse outcomes of interest (i.e., Tuckey et al., 2015; Espedido and Searle, 2018; Brady and Cunningham, 2019). However, and based on the JD-R model, we argue that an exclusive focus on job demands might not only be inconsistent from a theoretical point of view, but might also fail to provide an accurate understanding of the associations between job demands and work-related outcomes. Central to the JD-R model is the proposition that working conditions can be broadly understood as job resources or job demands (Bakker and Demerouti, 2017). Both favorable and unfavorable job characteristics are not only presumed to co-exist at the workplace, but are also expected to influence each other (Bakker and Demerouti, 2017). In general, the relations between job demands and work-related outcomes are expected to change, once job resources are taken into consideration (Van den Broeck et al., 2010). In this regard, job resources are not only posited to aid employees by buffering job demands, but also to enable them to attain other resources (Bakker and Demerouti, 2017). In a similar vein, the Conservation of Resource Theory (COR; Hobfoll, 2001) postulates that individuals are highly prompted to retain and to accumulate resources, which further corroborates the importance of taking into account job resources when conducting research on work-related stressors. Given that the JD-R model mandates a joint focus on job demands *and* job resources, we argue that the examination of job resources, next to the different types of job demands, namely challenges, hindrances, and threats, might provide a more accurate understanding of the presumed stressor-strain relations at work. As such, the present work aimed to examine the novel C-H-T distinction of workplace stressors (Tuckey et al., 2015), while considering the concept of job resources. In other words, we aimed to investigate the effects of challenges, hindrances, and threats on employees’ well-being, while controlling for the effects of job resources.

### 2.2. Research hypotheses

According to O’Brien and Beehr (2019) the use of classification systems (i.e., *a priori* categorizations) in the context of occupational stress primarily serves to summarize a wide range of individual stressors and their effects on work-related outcomes. Given that workplace stressors of the same category are presumed to have the same effects on employees, there is no substantial need to extensively examine the individual effects each stressor would have independently (O’Brien and Beehr, 2019). Accordingly, the use of categorization frameworks allows scholars to shift their focus of attention toward the examination of distinct stressor types and their differing relations to work-related variables. Indeed, empirical findings support the notion that workplace stressors, which correspond to different categories, differ as regards to their associations to work-related outcomes (O’Brien and Beehr, 2019). For instance, job challenges were found to positively relate to job satisfaction, motivation, and performance, whereas job hindrances yielded the reversed pattern of associations (Podsakoff et al., 2007; Van den Broeck et al., 2010). Note that although both challenges and hindrances showed positive relations with ill-being outcomes (e.g., emotional exhaustion; Podsakoff et al., 2007), hindrance stressors tend to relate more strongly to negative outcomes, than challenge stressors (e.g., burnout; Tuckey et al., 2015; O’Brien and Beehr, 2019). Moreover, job threats are presumed to result in particularly detrimental consequences (e.g., health problems and burnout; Nielsen and Einarsen, 2012; Tuckey et al., 2015). Accordingly, it is expected that threats relate more strongly to negative outcomes, than hindrance stressors. Therefore, examining the distinctiveness of different demand categories based on their associations to well-being outcomes might help us to gain a more simplified understanding of the occupational stress process.

Accordingly, the first aim of the present work was to test the distinctiveness of demand categories by examining their effects to psychological health variables (i.e., burnout, vigor).^1^ That is, we aimed to investigate the correlational structure of diverse stressor types by focusing on the stressor-strain associations, that is, the presumed effects of challenges, hindrances, and threats. In doing so, we focused on well-known workplace stressors, which have been discussed by researchers as regards to their *a-priori* classification into different demand categories (i.e., top-down approach; Crawford et al., 2010; Van den Broeck et al., 2010; Tuckey et al., 2015). Specifically, we have decided to focus on workplace mobbing, emotional demands, role ambiguity, job insecurity, time pressure and mental demands due to their typicality for a given type of demand (e.g., Crawford et al., 2010; Van den Broeck et al., 2010). Based on theoretical developments and empirical findings, we expected workplace mobbing and emotional demands to correspond to the category of job threats (Tuckey et al., 2015). In line with previous research, we expected role ambiguity and job insecurity to correspond to the category of job hindrances (Cavanaugh et al., 2000; Van den Broeck et al., 2010), and mental demands and time pressure to belong to the category of job challenges (Cavanaugh et al., 2000; Podsakoff et al., 2007; Van den Broeck et al., 2010). As regards to the selected job resources, we focused on autonomy and social support, as both are pertinent examples of this category (Van den Broeck et al., 2010). To examine the correlational structure of these different stressor types, while simultaneously accounting for the effects of job resources, we examined the C-H-T framework in relation to concurrent and widely used theoretical frameworks of job demands, such as the early JD-R model (Demerouti et al., 2001) or the amended JD-R model (Bakker and Demerouti, 2017). That is, we sought to identify which of these competing theoretical frameworks might best reflect the nature of job demands. In particular, and based on Tuckey et al. (2015) initial study, as well as on O’Brien and Beehr’s (2019) reasoning mentioned above, we expected that demands belonging to the categories of threats, hindrances, and challenges will differ as regards to their effects on well-being outcomes, but that those demands belonging to the same category would yield equal effects. Specifically, we hypothesized that:

*H1*: A model, in which the effects of demands corresponding to threats, hindrances and challenges is allowed to differ (while setting the effects of demands belonging to the same category to be equal) provides a better fit to the data than a model, in which all demands have equal effects on well-being outcomes (i.e., early JD-R model), or a model, in which the effects of demands corresponding to threats and hindrances is set to be the same, yet to differ from the effects of challenges (i.e., amended JD-R model).

To further examine the associations between different job characteristics and psychological health variables (i.e., burnout and vigor)^2^, we formulated four additional hypotheses. To develop our hypotheses, we drew from previous theoretical developments regarding the C-H-T framework (e.g., Tuckey et al., 2015), the revised JD-R model (e.g., Bakker and Demerouti, 2017), as well as related empirical findings (e.g., Van den Broeck et al., 2010; Nielsen and Einarsen, 2012; Tuckey et al., 2015). Specifically, we hypothesized that:

*H2*: Job threats positively relate to burnout (i.e., showing the strongest effects; *H2a*), but-negatively relate to vigor (*H2b*).

*H3*: Job hindrances positively relate to burnout (*H3a*), but negatively relate to vigor (*H3b*).

*H4*: Job challenges positively relate to burnout (H4a) and positively relate to vigor (*H4b*).

*H5*: Job resources negatively relate to burnout (H5a), but positively relate to vigor (*H5b*).

The second aim of the present work was to explore the interactive effects proposed by the JD-R model (Bakker and Demerouti, 2007, 2017). According to the JD-R model, job resources (a) will buffer the effect of job demands on work-related strain, and (b) will specially affect work-engagement when levels of job demands are high (Bakker and Demerouti, 2007, 2017). Although there has been some support for the joint effects of job demands and job resources on well-being outcomes (e.g., Bakker et al., 2005a, 2007, 2010), findings have not always been consistent (van den Tooren and de Jonge, 2008; Hu et al., 2011; O’Brien and Beehr, 2019; Gonzalez-Mulé et al., 2021). Moreover, it is not clear whether resources might moderate the relation between workplace stressors and strain differently, depending on the nature of the demand (O’Brien and Beehr, 2019). To the best of our knowledge, no study adopting the C-H-T distinction has yet investigated the presumed moderating relations between demands assigned to these different stressor categories and resources. Therefore we decided to adopt an exploratory approach by formulating the following research question:

*RQ1*: Do the presumed moderations between job resources and job demands differ depending on the type of demand (i.e., threat, hindrance, challenge)?

## 3. Materials and methods

### 3.1. Data collection

We conducted the present study within the framework of an extensive research project on quality of work in Luxembourg (Sischka and Steffgen, 2017). The project was carried out by the University of Luxembourg in cooperation with the Luxembourg Chamber of Labor. Data for the current study were collected in 2016 *via* Computer-Assisted Telephone Interviews (CATI) with employees working in Luxembourg. The survey was conducted following the guidelines of the Declaration of Helsinki (i.e., voluntary participation, right to withdraw consent at any time throughout the phone interviews without negative consequences). Prior start to the survey, informed consent was obtained from all participants verbally on the phone. Participants could choose between one of the following languages: Luxembourgish, French, German, or Portuguese. The language versions exhibited scalar measurement invariance (Steffgen et al., 2020). Data presented in the current study are cross-sectional.

### 3.2. Participants

A total of 1,506 employees working in Luxembourg (54.1% male, *n* = 815) participated in the study (Sischka and Steffgen, 2017). While 60.2% of participants indicated to live in Luxembourg (*n* = 906), 20.3% indicated to live in France (*n* = 305), 10.2% in Belgium (*n* = 153), and 9.4% in Germany (*n* = 142). The employees’ age ranged from 16 to 66 years (*M* = 45.8, SD = 8.9). The majority worked as academic professionals (26.4%, *n* = 397), as technicians and associate professionals (25.1%, *n* = 378), as clerical support workers (12.7%, *n* = 192), and others (35.8%, *n* = 529).

### 3.3. Measures

#### 3.3.1. Job characteristics

The Quality of Work Index (QoW) and the Quality of Employment Index (QoE; Steffgen et al., 2020) were administered to assess a range of different job characteristics. *Emotional demands* were measured by two items (e.g., “How often does your work require you to control your feelings?,” *ω* = 0.79). *Workplace mobbing* was measured with the Luxembourg Workplace Mobbing Scale (LWMS; Steffgen et al., 2019; Sischka et al., 2020) consisting of five items (e.g., “How often is your work criticized by your colleagues or your superior?,” *ω* = 0.73). For both dimensions, employees had to report how often they come across each situation on a 5-point Likert scale, ranging from 1 (= *never*) to 5 [= (*almost*) *always*]. *Job insecurity* was assessed with two items (e.g., “To what extent are you afraid to lose your job?”; ω = 0.76). Likewise, *role ambiguity* consisted of two items (e.g., “To what extent are your work tasks clearly defined?”; *ω* = 0.73). Both items were reversed to represent role ambiguity. For both dimensions, we employed a 5-point Likert response format ranging from 1 (= *to a very low extent*) to 5 (= *to a very large extent*). *Mental demands* was assessed with two items (e.g., “To what extent does your work demand concentration?”; *ω* = 0.74). *Time pressure* consisted of two items (e.g., “How often are you under time pressure or rushed in your work?”; *ω* = 0.73). Participants were asked to report how often they come across each of the described situation on a on a 5-point Likert scale, ranging from 1 (= *never*) to 5 [= *(almost) always*]. *Social support* was assessed with three items (e.g., “To what extent are you supported in your work by your colleagues?”; *ω* = 0.81). Finally, *Autonomy* consisted of four items (e.g., “To what extent can you decide how you carry out your work?”; *ω* = 0.76). Responses were made on a 5-point Likert scale, ranging from 1 (= *to a very low extent*) to 5 (= *to a very large extent*).

#### 3.3.2. Work-related burnout

Burnout was measured with six items of the work-related subscale of the Copenhagen Burnout Inventory (Kristensen et al., 2005). We modified the original wording of the items to fit the context of a telephone interview. An example item is “To what extent do you feel burn out by your work?”; (*ω* = 0.85). A 5-point Likert scale ranging from 1 (= *never/to a very low extent*) to 5 [= *(almost) always/to a very large extent*] was administered.

#### 3.3.3. Vigor

We employed the three-item vigor subscale of the shortened version of the Utrecht Work Engagement Scale (UWES-9; Schaufeli et al., 2006). We adapted the original wording of the items to fit the context of a telephone interview better (e.g., “How often do you have the feeling that you are overflowing with energy at work?”; *ω* = 0.71). Employees responded on a 5-point Likert scale, ranging from 1 (*= never*) *to 5* [*= (almost) always*].

### 3.4. Statistical analysis

First, we analyzed means, standard deviations, skewness, kurtosis, and correlations between the constructs of interest. Second, we performed confirmatory factor analysis (CFA) to develop a measurement model of job characteristics. Based on the distributional qualities of the data, we decided to use a maximum likelihood estimation with robust standard errors and scales test statistics (MLR; Yuan and Bentler, 2000). To determine each construct’s latent mean and variance in a non-arbitrary metric, we decided to apply the effects coding method for scale setting (Little et al., 2006). To evaluate the fit of the proposed measurement model, we considered frequently reported fit statistics. For the Comparative Fit Index (*CFI*), values of 0.90 are acceptable, whereas values of 0.95 or higher are indicative of good fit (Little, 2013). For the Tucker-Lewis Index (*TLI*), values of 0.95 or higher are indicative of excellent fit (Schreiber, 2008). For the Root Mean Square Error of Approximation (*RMSEA*) values up to 0.06 (with confidence interval of 0.00–0.08) and for Standardized Root Mean Square Residuals (*SRMR*) values of less than.08 indicate a good fit (Schreiber, 2008). Third, we investigated the relations between our independent variables (i.e., job characteristics) and the outcomes of interest (i.e., burnout, vigor) by performing structural equation modeling (SEM). To test the distinctiveness of the theoretically derived demand categories, we defined a series of structural models and compared them with each other.^3^ We used the Satorra-Bentler scaled *χ*2 difference test (Satorra, 2000), as well as the Akaike Information Criterion (*AIC*) and the Bayesian Information Criterion (*BIC*) to evaluate differences in fit between the structural models (Schreiber, 2008). Finally, we estimated interactions between latent variables by applying the two-steps estimation procedure of the latent moderated structural equation method (LMS; Klein and Moosbrugger, 2000; Maslowsky et al., 2015). In total, we estimated 12 interaction terms per outcome variable (i.e., 24 interactions). Statistical analyzes were done with IBM SPSS statistics (version 25) and Mplus (version 8.3; Muthén and Muthén, 2017). The Mplus code for all models can be obtained from the Supplementary material.

## 4. Results

### 4.1. Descriptive statistics and correlational analysis

Table 1 shows means, standard deviations and inter-correlations between the latent variables. The data were slightly skewed (univariate skewness−0.69 to 0.95) and kurtotic (univariate kurtosis−0.92 to 1.37), but below the absolute values of 2 and 7, respectively (Schreiber, 2008). When inspecting the correlations for multicollinearity, no relations above 0.80 were identified. Threats (i.e., emotional demands, mobbing) correlated positively with hindrances (i.e., role ambiguity, job insecurity) and with challenges (i.e., mental demands, time pressure). Both hindrances correlated positively with time pressure, yet mental demands and role ambiguity correlated negatively. Job resources (i.e., autonomy, social support) correlated negatively with threats, as well as with hindrances.

**Table 1:** Means, standard deviations, and inter-correlations among latent variables.

Variable	*M*	SD	1	2	3	4	5	6	7	8	9	10
1. Workplace mobbing	1.83	0.48	_									
2. Emotional demands	3.03	1.03	0.42***	_								
3. Job insecurity	2.08	0.84	0.33***	0.17***	_							
4. Role ambiguity	2.24	0.64	0.44***	0.14***	0.30***	_						
5. Time pressure	3.50	0.80	0.32***	0.52***	0.23***	0.11**	_					
6. Mental demands	3.98	0.60	0.12**	0.35***	0.00	−0.17***	0.50***	_				
7. Autonomy	3.24	0.74	−0.37***	−0.21***	−0.29***	−0.27***	−0.26***	0.04	_			
8. Social support	3.84	0.68	−0.41***	−0.11**	−0.29***	−0.36***	−0.10**	0.12**	0.31***	_		
9. Burnout	2.43	0.71	0.62***	0.50***	0.38***	0.37***	0.44***	0.22***	−0.35**	−0.29***	_	
10. Vigor	3.42	0.67	−0.46***	−0.23***	−0.37***	−0.34***	−0.21***	0.00	0.32***	0.33***	−0.69***	_

*p < 0.05. **p < 0.01. ***p < 0.001.

### 4.2. Factor analysis

We performed a first-order confirmatory factor analysis (CFA) to develop a measurement model of job characteristics (i.e., independent measures). For latent variables that consisted of only two items, we have equated the respective factor loadings of the items to prevent estimation problems (i.e., Heywood cases)^4^. The fit indices of the measurement model of job characteristics indicated a good fit of the data to the hypothesized structure (*∆χ^2^* = 511.587, *∆df* = 186, *p* < 0.001, *RMSEA* = 0.034 (0.031; 0.038), *SRMR* = 0.039, *CFI* = 0.956, *TLI* = 0.946), supporting the predicted measurement model of job characteristics.

### 4.3. Main effects

To test the relations between different job characteristics and employees’ well-being (H1–H5), we estimated a structural model. We modeled job characteristics belonging to the categories of threats, hindrances, challenges and resources as concurrent predictors of vigor and burnout.^5^ To test the distinctiveness of the proposed demand categories, we compared our hypothesized structural model (Model C) with two alternative models (Model A and B). Model C reflects the C-H-T distinction of job demands, in which the effects of job demands corresponding to challenges, hindrances and threats on well-being outcomes are expected to differ, yet the effects of demands belonging to the same category are presumed to be equal. That is, we allowed the effects of threats, hindrances, and challenges to differ from each other, and we constrained the path coefficients between those demands corresponding to the same category and well-being outcomes to be equal to each other. In contrast, Model A reflects the early JD-R model, in which all job demands are expected to belong to one overall demand category, hence, yielding equal effects on well-being outcomes. Therefore, we constrained the path coefficients between all demands and psychological health variables to be equal. Contrary to Model A and Model C, Model B reflects the amended JD-R model, in which job demands are expected to be classified into two categories (i.e., challenges and hindrances). Note that in this model the category of hindrances also comprises demands, which have been referred to in later theoretical developments as threats. Specifically, we constrained the path coefficients between workplace mobbing, emotional demands, job insecurity, and role ambiguity on the one hand, and vigor and burnout, on the other hand, to be equal. In addition, we constrained the path coefficients between time pressure and mental demands (i.e., challenges) on the one hand, and vigor and burnout, on the other hand, to be equal. Hence, in Model B, threats and hindrances are assumed to yield equal effects on employees’ well-being, yet to differ from the effects of challenges.

**Figure 1:**
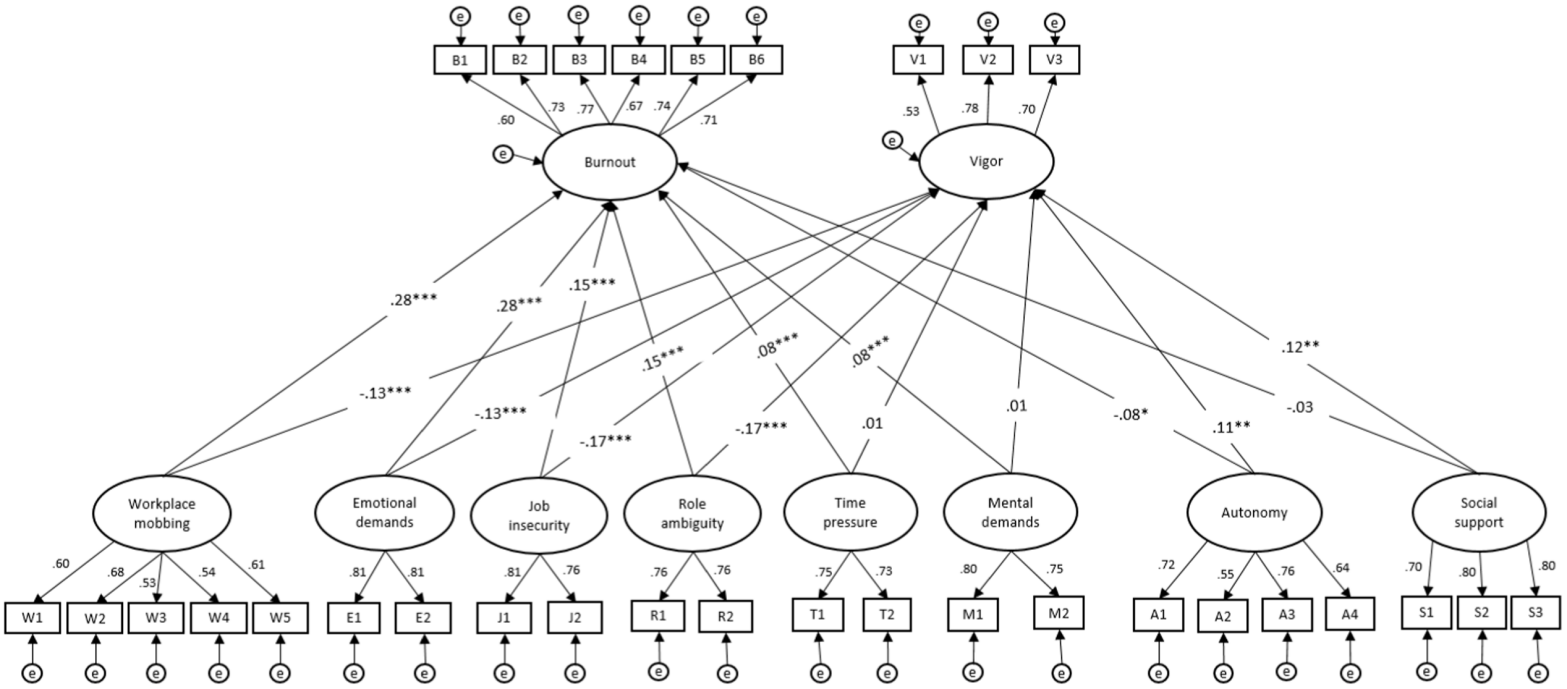
Structural model of the relationships between job threats, job hindrances, job challenges, job resources, and burnout and vigor (Model C). This structural equation model predicts employees’ burnout and vigor from threats, hindrances, challenges and job resources. *χ*^2^(400) = 1341.773, RMSEA = 0.040 (0.037; 0.042), SRMR = 0.043, CFI = 0.927, TLI = 0.915. Coefficients represent standardized estimates. *p < 0.05. **p < 0.01. ***p < 0.001.

As can be seen in Table 2, Model B shows an improved model fit relative to Model A. Moreover, specifying the path coefficients between threats and hindrances to psychological health variables to be different from each other in Model C further improved model fit. Indeed, the fit indices of the hypothesized model (i.e., Model C) indicate a good fit of the data to the hypothesized structure. In addition, Model C was characterized by the lowest *AIC* and *BIC* values, further supporting a better fit to the data compared to the alternative models. Moreover, the computed chi-square difference tests between (1) Model A and Model C (*∆χ^2^* = 61.88, ∆df = 4, *p* < 0.001), and between (2) Model B and Model C (*∆χ^2^* = 25.42, ∆df = 2, *p* < 0.001), were significant, illustrating that the hypothesized solution fits the data better than the alternative models. Overall, these results support the distinctiveness of the proposed threefold demand classification in terms of their effects, allowing to maintain our hypothesis 1. Figure 1 illustrates the final SEM model (i.e., Model C) and provides standardized values, as well as respective fit indices.

**Table 2:** Comparison of fit indices of various SEM models of job characteristics on well-being.

Model	Description	*χ* ^2^			RMSEA		SRMR	CFI	TLI	AIC	BIC
		Value	df	*p*	Value	90%CI					
A	Path coefficients between all job demands and well-being outcomes constrained to be equal.	1399.537	404	0.000	0.040	(0.038; 0.043)	0.047	0.923	0.911	114237.824	114891.841
B	Path coefficients between threats and well-being outcomes constrained to be equal to path coefficients between hindrances and well-being outcomes; yet to differ from path coefficients between challenges and well-being outcomes.	1365.282	402	0.000	0.040	(0.038; 0.042)	0.044	0.925	0.913	114202.112	114866.763
C	Path coefficients between threats and well-being outcomes constrained to be equal; yet to differ from path coefficients between (a) hindrances to well-being outcomes, and (b) challenges to well-being outcomes.	1341.773	400	0.000	0.040	(0.037; 0.042)	0.043	0.927	0.915	114179.263	114854.549

MLR estimator. RMSEA, Root Mean Square Error of Approximation; SRMR, Standardized Root Mean Square Residuals; CFI, Comparative Fit Index; TLI, Tucker-Lewis Index; AIC, Akaike Information Criterion; BIC, Bayesian Information Criterion.

In line with hypothesis 2a, both job threats (i.e., emotional demands, workplace mobbing) had the strongest positive effects on burnout (*β* = 0.28, *p* < 0.001). In line with hypothesis 2b, both threats were negatively related with vigor (*β* = −0.13, *p* < 0.001). Conforming to hypothesis 3a, job hindrances (i.e., job insecurity, role ambiguity) had a significant positive effect on burnout (*β* = 0.15, *p* < 0.001). Moreover, both hindrances predicted vigor (*β* = −0.17, *p* < 0.001), supporting hypothesis 3b. In line with hypothesis 4a, both job challenges (i.e., time pressure, mental demands) had a significant positive effect on burnout (*β* = 0.08, *p* < 0.001). However, results failed to support the expected relation between both job challenges and vigor (H4b, *β* = 0.01, *p* > 0.05) after controlling for other work characteristics. Finally, hypothesis 5a was partly confirmed. Whereas autonomy had a negative relation to burnout (*β* = −0.08, *p* < 0.05), social support did not relate to the latter (*β* = −0.03, *p* > 0.05). In line with hypothesis 5b, both types of job resources had a significant positive effect on vigor (for autonomy: *β* = 0.11, *p* < 0.01; for social support: *β* = 0.12, *p* < 0.01). Overall, the model explained 51.2% of the variance in burnout and 28.5% of the variance in vigor.

### 4.4. Interactive effects

To explore possible interaction effects between different job demands and job resources on employees’ well-being (RQ1), we estimated latent variables interactions within a SEM framework. Results of the analysis yielded a log-likelihood difference value of *D* = 29.328, with df = 24. Using a *χ*2 distribution, this log-likelihood test proved non-significant (*p* > 0.05), which indicates that the null model (i.e., comprising only main effects) does not represent a significant loss in fit relative to the alternative model (i.e., Model 1: comprising main effects and interaction terms). Therefore, the model including interaction terms was rejected.

## 5. Discussion

The goal of the present study was to advance our understanding on the nature of job demands and their associations with employees’ well-being by examining Tuckey et al. (2015) proposed Challenge-Hindrance-Threat distinction of workplace stressors, while taking into account the effects of job resources. Overall, results of the present study supported the distinctiveness of job demands in terms of their effects. Among a large and heterogeneous sample of occupations, the threefold differentiation of job demands provided a better fit to the data than a one-fold (i.e., reflecting the early JD-R model) or a two-fold differentiation of job demands (i.e., reflecting the amended JD-R model). Notably, results supported the presumed health impairing effects of both job threats (Tuckey et al., 2015; Searle and Tuckey, 2017). Consistent with our hypotheses, both job hindrances related to employees’ well-being in an equivalent manner. As regards to job challenges, our understanding about their nature and psychological effects is perhaps less clear. Whereas both job challenges predicted burnout, they did not predict vigor, and thus, failed to contribute to optimal psychological functioning. Clearly, these findings contrast previous studies conducted within the JD-R literature, which typically indicate positive associations between job challenges and (components of) work engagement (Bakker et al., 2005b; Mauno et al., 2007). Yet, rather than focusing on the effects of challenges in relation to employees’ sense of dedication (as in Tuckey et al., 2015), we examined their potential to predict employees’ experience of vigor. Although both constructs represent subcomponents of work engagement, they differ as regards to their definitions and related effects. Dedication refers to the actual experience of challenge, whereas vigor refers to an energetic state (Schaufeli et al., 2006). Therefore, it might be plausible that the stimulation of employees’ dedication may more accurately describe the nature of job challenges by its mere definition, than an enrichment in terms of employees’ energetic levels.

Regarding LMS analyzes, results indicated that the inclusion of interactive terms within the analytical framework did not contribute to an enhanced explanation of the relations between job characteristics and employees’ well-being. This finding contrasts previous research conducted on the JD-R model, in which the moderating effects of demands and resources on job related outcomes have been proposed (e.g., Bakker et al., 2005a). A plausible explanation might be an existing mismatch between our chosen job demands and job resources (Hu et al., 2011). Job resources and job demands do not randomly interact with each other (van den Tooren and de Jonge, 2008). Rather, the joint effects on job related outcomes are presumed to emerge if job demands and job resources coincide in terms of their specific components (van den Tooren and de Jonge, 2008). Yet, the present study encompassed a more global focus, as we considered job characteristics from several areas in a simultaneous manner, which in turn might have masked matching interactions.

### 5.1. Theoretical contributions

The present study contributes to the literature on the JD-R model and existing stressor frameworks as follows. First, it incorporates the most recent distinction of workplace stressors (Tuckey et al., 2015) into the JD-R model (Bakker and Demerouti, 2017). In doing so, the present study followed LePine’s (2022) recent suggestion to acknowledge the concept of threats when conducting research on workplace stressors. In addition, the current study addresses O’Brien and Beehr’s (2019) call to combine multiple theoretical frameworks, seeking to accumulate knowledge on psychological phenomena. Indeed, extending the basic tenants of the JD-R model by incorporating the C-H-T distinction allows us to develop a more differentiated understanding on the nature of job demands, and in particular, as regards to their associated effects on employees’ well-being. Findings of the present study corroborate the importance of differentiating between job demands in terms of their effects (i.e., predictions). Instead of modeling different job demands as indicators of a common factor, we followed Hayduk (2014) recommendation to introduce theorized latent-level effects of factors, as it provides a robust assessment of the factors’ casual structuring. Most notably, contemplating challenges, hindrances and threats based on their associations to well-being outcomes might constitute a useful practice, as it simplifies the breadth of existing stressor-strain associations, and it might allow researchers to estimate the effects of individual stressors in advance. Moreover, the present study complements Tuckey et al. (2015) research by addressing its shortcoming to (statistically) consider job resources when examining the associations between different types of job demands and psychological health outcomes. As such, and based on the tenants of the JD-R model, the present study provides a more theoretical consistent account of the associations between job demands and work-related outcomes. In addition, including job resources into our analytical framework allowed us to revise the key propositions of the JD-R model, providing a robust validation among a representative sample of the Luxemburgish working population.

### 5.2. Practical implications

Our results have important implications for the workplace. Given that findings of the present study have demonstrated the health impairing nature of job challenges, but have failed to support their presumed stimulating effects, we advise employers who wish to promote their employees’ mental energy to implement certain job redesign techniques with caution. While some employers may intent to stimulate employees through a focus on job challenges (e.g., time pressure), this specific approach might backfire. Indeed, results from a recent meta-analysis have shown that job challenges may not always provide benefits to employees and organizations (Mazzola and Disselhorst, 2019). Therefore, focusing on job resources and on their unique motivational effects, may constitute a more secure and effective practice for the development of employees’ flourishing. Indeed, boosting resources is considered a leading measure to improve employee engagement (Mazzetti et al., 2021). To give an illustration, a study examining the effectiveness of a job-resources based intervention found that employees, who reported an initial level of work-engagement, were successful in building their team innovativeness and stayed engaged (Seppälä et al., 2018). Lastly, and given that findings of the current study support Tuckey et al. (2015) idea to differentiate hindrances from threats (in terms of their effects), even when job resources are being considered, we agree with Tuckey et al. (2015) and advise occupational health practitioners to focus on threatening demands, in addition to hindering and challenging demands, when planning and carrying out primary stress prevention programs at the workplace. For instance, threatening demands, such as emotional demands, could be prevented by adequate training (Tuckey et al., 2015).

### 5.3. Study limitations and recommendations for future studies

Although the present study contributes to research on the JD-R model and existing stressor frameworks, some limitations need to be addressed. First, researchers might argue that the concept of stressor appraisal is being excluded from the current investigation (e.g., Brady and Cunningham, 2019). In this regard, scholars might outline that the use of classification frameworks may not always accurately depict how stressors are being understood and perceived by individuals (e.g., Brady and Cunningham, 2019). Although we acknowledge this line of reasoning, the present study is based on the premises of the JD-R model, which generally assume the existence of objectives differences between distinct types of job characteristics (Van den Broeck et al., 2010; Bakker and Demerouti, 2017). Besides, we have decided to examine the novel C-H-T model through a resource perspective, as job resources have been referred to as the defining features to understand stress (O’Brien and Beehr, 2019). Nevertheless, future research could take into account the concept of stressor appraisal when examining individual differences within the stress process.

Second, the present study is based on a cross-sectional design. Therefore, no conclusions about causality can be drawn. Note, however, that the purpose of the present study was not to establish a casual order between the study variables, but to examine the associations between job demands and employees’ well-being, while taking into account job resources. Given that a cross-sectional research design is the method of choice if the researchers’ intention is to investigate associations between variables, and in particular, when seeking to examine new concepts in old domains of research (Spector, 2019), we consider our decision to use a cross-sectional design appropriate for the context of the present study. In addition, the model aligns with theoretical accounts on the relations between job characteristics and employees’ well-being (e.g., Bakker and Demerouti, 2017), as well as with previous studies implementing longitudinal designs (e.g., Hakanen et al., 2008; Tuckey et al., 2015). Notwithstanding, futures studies might use (quasi-) experimental designs (e.g., job redesign interventions) to uncover the causative mechanisms linking the four types of job characteristics with employees’ well-being.

Third, the current study relied exclusively on self-report measures, which are likely to be affected by subjective bias from the respondents. In this regard, scholars might raise concerns regarding the use of single source, self-report measures in terms of common method variance (CMV). As such, employing a common measurement method might impact the associations between the study constructs, resulting in bias, if the method, as a casual factor, systematically confounds the relationships among variables (Fuller et al., 2016; Williams and McGonagle, 2016). Note, however, that the issue of common method variance in organizational research has been overstated (Spector, 2006; Conway and Lance, 2010). Indeed, empirical evidence has led scholars to question the myth that the nature of the method itself may lead to inflated correlations (Spector, 2006). Moreover, a recent review suggested that there is a low probability for CMV to invalidate research findings (Bozionelos and Simmering, 2022). In a similar vein, results from a simulation study suggested that CMV does not inevitably constitute a threat to the validity of results (Fuller et al., 2016). As scale reliability is argued to influence the degree to which CMV leads to bias, research characterized by single-item measures and reporting very high to perfect reliabilities is more likely to be prone to common method bias (Fuller et al., 2016). In the context of the present study, reliability analysis resulted in coefficients ranging from 0.71–0.85, suggesting rather low to typical reliabilities (see Fuller et al., 2016), and thus, lower risk of CMV confounding the findings. Nevertheless, future studies might consider multiple sources to assess the constructs of interest (Spector, 2019), as well as integrate objective measures (e.g., physiological measures) to further examine employees’ health, providing objective data.

## 6. Conclusion

To conclude, distinguishing between threats, hindrances, and challenges, along job resources constitutes an important extension of the JD-R model. Results have demonstrated that demands corresponding to the categories of threats, hindrances, and challenges differ in terms of their effects, and can therefore be treated as separate dimensions of workplace stressors. Our analyzes provided strong support for the general health impairing nature of job demands. Whereas job threats and job hindrances might be best characterized by their potential to function in a health and motivational impairing manner, with job threats relating more strongly to burnout and job hindrances associating more strongly with vigor, job challenges did not associate with employees’ experience of vigor. However, strong support was found for the motivational potential of job resources. Therefore, examining threats, along hindrances, challenges, as well as resources should promote a more fine-grained understanding of employees’ wellbeing. Based on these results, we suggest incorporating the threefold differentiation of workplace stressors to advance the JD-R model.

## Data availability statement

The raw data supporting the conclusions of this article will be made available by the authors, without undue reservation.

## Ethics statement

Ethical review and approval was not required for the study on human participants in accordance with the local legislation and institutional requirements. Written informed consent for participation was not required for this study in accordance with the national legislation and the institutional requirements.

## Author contributions

MFH, PS, and GS wrote the manuscript and were responsible for research design and ideas. PS and GS were responsible for developing the questionnaire, collecting the data and research revision. MFH was responsible for analyzing the data. All authors contributed to the article and approved the submitted version.

## Funding

This work was supported by a grant from the Luxembourg Chamber of Labor.

## Conflict of interest

The authors declare that the research was conducted in the absence of any commercial or financial relationships that could be construed as a potential conflict of interest.

## Publisher’s note

All claims expressed in this article are solely those of the authors and do not necessarily represent those of their affiliated organizations, or those of the publisher, the editors and the reviewers. Any product that may be evaluated in this article, or claim that may be made by its manufacturer, is not guaranteed or endorsed by the publisher.

## Footnotes

^1^Note that previous research conducted on the C-H-T framework has primarily focused on its distinction in terms of dimensions (i.e., measurement level). In doing so, researchers have often relied on item parceling procedures when examining the correlational structure of job demands (e.g., Tuckey et al., 2015). Yet, this practice is problematic due to its potential to hide misspecification errors and possible bias in parameter estimates (Marsh et al., 2013). Although stressors assigned to one category are expected to be similar in meaning, this does not "certify dependence of those indicators on a common cause" (Hayduk, 2014, p. 907). Moreover, modeling indicators as indicators of a common factor encompasses merely the joint variance among them, failing to grasp the unique facets of its components (Rhemtulla et al., 2020). Given that threat stressors may derive from qualitative different areas of work, and hence, carry unique features, we argue that contemplating stressor categories in terms of an underlying common cause (i.e., on the dimensional level: a common factor) might be inappropriate.

^2^In line with previous research examining the effects of workplace stressors (i.e., challenge, hindrance/threat), we decided to focus on burnout and vigor as outcome variables (e.g., Van den Broeck et al., 2010; Tuckey et al., 2015).

^3^Note, that our defined models do not capture the full complexities of the JD-R model (nor its developments). Instead, our aim was to focus on one excerpt of the model, as it is not feasible to investigate the JD-model in its entirety.

^4^This technique is based on the assumption that the constructs are represented by tau equivalent items. Tau-equivalent indicators are defined as indicators of a construct that are equivalent in terms of their loadings, but may vary in terms of their intercepts (Little, 2013, p. 34).

^5^The residuals of vigor and burnout were allowed to correlate. Correlating error terms between the outcome variables is reasonable, if one hypothesizes that they share some amount of variance that is not explained by the predictors. Several meta-analyzes have shown that our employed outcome variables are highly correlated (e.g., Halbesleben, 2010).
