# Supplementary material for: Examining the challenge-hindrance-threat distinction of job demands alongside job resources
Source: Front Psychol. 2023 Feb 17;14:1011815. doi: 10.3389/fpsyg.2023.1011815 (PMC9983325; doi:10.3389/fpsyg.2023.1011815)
Supplement: Supplementary file 1 [file Data_Sheet_1.docx]

# Supplemental material

*Mplus Code for the measurement model of job characteristics*

TITLE: measurement model of job characteristics

DATA: file = “file name”;

VARIABLE:

Names = W1 W2 W3 W4 W5 E1 E2 R1 R2 J1 J2 T1 T2 M1 M2 A1 A2 A3 A4 S1 S2 S3;

Missing are all (96,97,98,99);

Usevariables = W1 W2 W3 W4 W5 E1 E2 R1 R2 J1 J2 T1 T2 M1 M2 A1 A2 A3 A4 S1

S2 S3;

ANALISIS:

type = general;

estimator = mlr;

MODEL:

!Defining first-order latent constructs

mobb BY W1* W2 W3 W4 W5;

ed BY E1* E2 (ed);

ra BY R1* R2 (ra);

jis BY J1* J2 (jis);

tp BY T1* T2 (tp);

md BY M1* M2 (md);

au BY A1* A2 A3 A4;

ss BY S1* S2 S3;

mobb@1; ed@1; jis@1; ra@1; tp@; md@; au@1; ss@1;

OUTPUT: sampstat standardized stdyx residual modindices tech4;

*Mplus Code for the structural equation model of job characteristics and employees’ well-being (i.e.,* ***Model A****)*

TITLE: SEM of job characteristics and well-being

DATA: file = “file name”;

VARIABLE:

Names = W1 W2 W3 W4 W5 E1 E2 R1 R2 J1 J2 T1 T2 M1 M2 A1 A2 A3 A4 S1 S2 S3 B1 B2 B3 B4 B5 B6 V1 V2 V3;

Missing are all (96,97,98,99);

Usevariables = W1 W2 W3 W4 W5 E1 E2 R1 R2 J1 J2 T1 T2 M1 M2 A1 A2 A3 A4 S1 S2 S3 B1 B2 B3 B4 B5 B6 V1 V2 V3;

ANALISIS:

type = general;

estimator = mlr;

MODEL:

!Defining first-order latent constructs

mobb BY W1* W2 W3 W4 W5;

ed BY E1* E2 (ed);

ra BY R1* R2 (ra);

jis BY J1* J2 (jis);

tp BY T1* T2 (tp);

md BY M1* M2 (md);

au BY A1* A2 A3 A4;

ss BY S1* S2 S3;

cbi BY B1* B2 B3 B4 B5 B6;

vig BY V1* V2 V3;

mobb@1; ed@1; jis@1; ra@1; tp@1; md@1; au@1; ss@1; cbi@1; vig@1;

!Specifying paths

cbi ON mobb ed ra jis tp md (1)

au ss;

vig ON au ss

tp md mobb ed ra jis (2);

!Specifying correlations

mobb WITH ed jis ra tp md au ss;

ed WITH jis ra tp md au ss;

jis WITH ra tp md ss au;

ra WITH tp md ss au;

tp WITH md ss au;

md WITH au ss;

au WITH ss;

cbi WITH vig;

OUTPUT: sampstat standardized stdyx residual modindices;

*Mplus Code for the structural equation model of job characteristics and employees’ well-being (i.e.,* ***Model B****)*

TITLE: SEM of job characteristics and well-being

DATA: file = “file name”;

VARIABLE:

Names = W1 W2 W3 W4 W5 E1 E2 R1 R2 J1 J2 T1 T2 M1 M2 A1 A2 A3 A4 S1 S2 S3 B1 B2 B3 B4 B5 B6 V1 V2 V3;

Missing are all (96,97,98,99);

Usevariables = W1 W2 W3 W4 W5 E1 E2 R1 R2 J1 J2 T1 T2 M1 M2 A1 A2 A3 A4 S1 S2 S3 B1 B2 B3 B4 B5 B6 V1 V2 V3;

ANALISIS:

type = general;

estimator = mlr;

MODEL:

!Defining first-order latent constructs

mobb BY W1* W2 W3 W4 W5;

ed BY E1* E2 (ed);

ra BY R1* R2 (ra);

jis BY J1* J2 (jis);

tp BY T1* T2 (tp);

md BY M1* M2 (md);

au BY A1* A2 A3 A4;

ss BY S1* S2 S3;

cbi BY B1* B2 B3 B4 B5 B6;

vig BY V1* V2 V3;

mobb@1; ed@1; jis@1; ra@1; tp@1; md@1; au@1; ss@1; cbi@1; vig@1;

!Specifying paths

cbi ON mobb ed ra jis (1)

tp md (2)

au ss;

vig ON au ss

mobb ed ra jis (3)

tp md (4) ;

!Specifying correlations

mobb WITH ed jis ra tp md au ss;

ed WITH jis ra tp md au ss;

jis WITH ra tp md ss au;

ra WITH tp md ss au;

tp WITH md ss au;

md WITH au ss;

au WITH ss;

cbi WITH vig;

OUTPUT: sampstat standardized stdyx residual modindices;

*Mplus Code for the structural equation model of job characteristics and employees’ well-being (i.e.,* ***Model C****)*

TITLE: SEM of job characteristics and well-being

DATA: file = “file name”;

VARIABLE:

Names = W1 W2 W3 W4 W5 E1 E2 R1 R2 J1 J2 T1 T2 M1 M2 A1 A2 A3 A4 S1 S2 S3 B1 B2 B3 B4 B5 B6 V1 V2 V3;

Missing are all (96,97,98,99);

Usevariables = W1 W2 W3 W4 W5 E1 E2 R1 R2 J1 J2 T1 T2 M1 M2 A1 A2 A3 A4 S1 S2 S3 B1 B2 B3 B4 B5 B6 V1 V2 V3;

ANALISIS:

type = general;

estimator = mlr;

MODEL:

!Defining first-order latent constructs

mobb BY W1* W2 W3 W4 W5;

ed BY E1* E2 (ed);

ra BY R1* R2 (ra);

jis BY J1* J2 (jis);

tp BY T1* T2 (tp);

md BY M1* M2 (md);

au BY A1* A2 A3 A4;

ss BY S1* S2 S3;

cbi BY B1* B2 B3 B4 B5 B6;

vig BY V1* V2 V3;

mobb@1; ed@1; jis@1; ra@1; tp@1; md@1; au@1; ss@1; cbi@1; vig@1;

!Specifying paths

cbi ON mobb ed (1)

ra jis (2)

tp md (3)

au ss;

vig ON au ss

mobb ed (4)

ra jis (5)

tp md (6) ;

!Specifying correlations

mobb WITH ed jis ra tp md au ss;

ed WITH jis ra tp md au ss;

jis WITH ra tp md ss au;

ra WITH tp md ss au;

tp WITH md ss au;

md WITH au ss;

au WITH ss;

cbi WITH vig;

OUTPUT: sampstat standardized stdyx residual modindices;

*Mplus Code for the latent moderated structural equation model (i.e. Model 0)*

TITLE: SEM of job characteristics and well-being

DATA: file = “file name”;

VARIABLE:

Names = W1 W2 W3 W4 W5 E1 E2 R1 R2 J1 J2 T1 T2 M1 M2 A1 A2 A3 A4 S1 S2 S3 B1 B2 B3 B4 B5 B6 V1 V2 V3;

Missing are all (96,97,98,99);

Usevariables = W1 W2 W3 W4 W5 E1 E2 R1 R2 J1 J2 T1 T2 M1 M2 A1 A2 A3 A4 S1 S2 S3 B1 B2 B3 B4 B5 B6 V1 V2 V3;

ANALISIS:

type = general;

estimator = mlr;

MODEL:

!Defining first-order latent constructs

mobb BY W1* W2 W3 W4 W5;

ed BY E1* E2 (ed);

ra BY R1* R2 (ra);

jis BY J1* J2 (jis);

tp BY T1* T2 (tp);

md BY M1* M2 (md);

au BY A1* A2 A3 A4;

ss BY S1* S2 S3;

cbi BY B1* B2 B3 B4 B5 B6;

vig BY V1* V2 V3;

mobb@1; ed@1; jis@1; ra@1; tp@1; md@1; au@1; ss@1; cbi@1; vig@1;

!Specifying paths

cbi ON mobb ed ra jis tp md au ss;

vig ON au ss tp md mobb ed ra jis;

!Specifying correlations

mobb WITH ed jis ra tp md au ss;

ed WITH jis ra tp md au ss;

jis WITH ra tp md ss au;

ra WITH tp md ss au;

tp WITH md ss au;

md WITH au ss;

au WITH ss;

cbi WITH vig;

OUTPUT: sampstat standardized stdyx residual modindices;

*Mplus Code for the latent moderated structural equation model (i.e. Model 1)*

TITLE: Latent SEM

DATA: file = “file name”;

VARIABLE:

Names = W1 W2 W3 W4 W5 E1 E2 R1 R2 J1 J2 T1 T2 M1 M2 A1 A2 A3 A4 S1 S2 S3 B1 B2 B3 B4 B5 B6 V1 V2 V3;

Missing are all (96,97,98,99);

Usevariables = W1 W2 W3 W4 W5 E1 E2 R1 R2 J1 J2 T1 T2 M1 M2 A1 A2 A3 A4 S1 S2 S3 B1 B2 B3 B4 B5 B6 V1 V2 V3;

ANALISIS:

type = random;

estimator = mlr;

algorithm = integration;

integration = montecarlo(5000);

MODEL:

!Defining first-order latent constructs

mobb BY W1* W2 W3 W4 W5;

ed BY E1* E2 (ed);

ra BY R1* R2 (ra);

jis BY J1* J2 (jis);

tp BY T1* T2 (tp);

md BY M1* M2 (md);

au BY A1 A2 A3 A4;

ss BY S1 S2 S3;

cbi BY B1* B2 B3 B4 B5 B6;

vig BY V1* V2 V3;

mobb@1; ed@1; jis@1; ra@1; tp@1; md@1; au@1; ss@1; cbi@1; vig@1;

!Defining interaction terms

auXmobb | au XWITH mobb;

auXed | au XWITH ed;

auXjis | au XWITH jis;

auXra | au XWITH ra;

auXtp | au XWITH tp;

auXmd | au XWITH md;

ssXmobb | ss XWITH mobb;

ssXed | ss XWITH ed;

ssXjis | ss XWITH jis;

ssXra | ss XWITH ra;

ssXtp | ss XWITH tp;

ssXmd | ss XWITH md;

!Specifying paths

cbi ON mobb ed ra jis tp md au ss auXmobb auXed auXjis auXra auXtp auXmd ssXmobb ssXed ssXjis ssXra ssXtp ssXmd;

vig ON au ss tp md mobb ed ra jis auXmobb auXed auXjis auXra auXtp auXmd ssXmobb ssXed ssXjis ssXra ssXtp ssXmd;

!Specifying correlations

mobb WITH ed jis ra tp md au ss;

ed WITH jis ra tp md au ss;

jis WITH ra tp md ss au;

ra WITH tp md ss au;

tp WITH md ss au;

md WITH au ss;

au WITH ss;

cbi WITH vig;

OUTPUT: sampstat standardized stdyx residual modindices;
